# Gaps in Diagnosis, Treatment, and Outcomes Among Patients with Brain Tumors in the United States: A State-of-the-Art Review

**DOI:** 10.3390/cancers17243982

**Published:** 2025-12-13

**Authors:** Vivek Podder, Zouina Sarfraz, Khalid Ahmad Qidwai, Arun Maharaj, Tulika Ranjan, Sonikpreet Aulakh, Manmeet S. Ahluwalia

**Affiliations:** 1 Mount Sinai Medical Center, Miami Beach, FL 33140, USA; vivek.podder@msmc.com; 2 Miami Cancer Institute, Baptist Health South Florida, Miami, FL 33176, USAkhalid.qidwai@baptisthealth.net (K.A.Q.);; 3 Dartmouth Hitchcock Medical Center and Clinics, Lebanon, NH 03756, USA; 4 West Virginia University, Morgantown, WV 26506, USA; 5 Herbert Wertheim College of Medicine, Florida International University, Miami, FL 33199, USA

**Keywords:** brain tumors, central nervous system tumors, healthcare access, socioeconomic factors, survival outcomes, diagnostic delay, geographic access, treatment equity

## Abstract

Brain tumors, both malignant and non-malignant, represent a persistent global health challenge. Differences in diagnosis, treatment, and outcomes are influenced by race, ethnicity, socioeconomic status (SES), and geographical location. Brain and central nervous system (CNS) tumors rank 19th in global cancer incidence and 12th in cancer-related mortality. U.S. Incidence is higher in females and individuals with greater socioeconomic means, contrasting with global patterns where males are more affected. Glioblastoma has a wide variation in incidence and survival by state, with rural regions showing higher mortality despite lower incidence, often due to reduced access to specialized care. Non-Hispanic Black children with CNS tumors experience higher mortality than their White peers, even after adjusting for SES. Outcomes are generally poorer in low- and middle-income countries, where healthcare infrastructure remains limited. Biological and genetic differences may also influence treatment response and tumor behavior across population groups. This review outlines key variations in brain tumor care, with a key focus on the United States, and emphasizes the need for patient-centered strategies to ensure timely diagnosis, consistent treatment, and improved outcomes.

## Simple Summary

Brain tumors are critical conditions that affect individuals of all age groups. Their diagnosis and treatment vary by geography, income, race and ethnicity. Delays in brain tumor diagnosis or treatment worsen outcomes. This review examines documented reasons for the socioeconomic and racial/ethnic differences in brain tumor diagnosis and treatment and explores how factors like health insurance, distance from medical centers, and the availability of appropriate clinical care can influence diagnosis, treatment, and outcomes. By better understanding these patterns in the United States, this review aims to identify ways to make brain tumor care more consistently accessible and help guide future research, healthcare policy, and clinical practice, to ensure that all patients, regardless of race, income, or urban/rural residence, have a fair chance at early diagnosis, effective treatment, and better survival.

## 1. Introduction

Brain tumors, both malignant and non-malignant, remain a global clinical and public health concern [1]. In the United States (US) alone, an estimated 24,820 malignant brain and spinal cord tumors will be diagnosed in 2025, and 18,330 individuals are expected to die from these tumors, according to the American Cancer Society 2025 update [2]. These estimates do not include benign tumors, which would substantially increase the overall burden. Treatment is typically multimodal, combining surgery, radiotherapy, chemotherapy, and, increasingly, targeted molecular and immunotherapeutic approaches, as well as emerging modalities such as tumor treating fields, CAR T-cell therapy, and oncolytic viruses [3].

Variation in diagnosis, treatment, and survival outcomes has been consistently observed across patient groups, influenced by race, ethnicity, socioeconomic status (SES), and geographic location [4,5]. In addition to tumor biology, survival may be influenced by insurance coverage, income level, level of attained education, and the structure of local healthcare systems [3,6,7]. Patients from lower-income locations or minority racial and ethnic backgrounds often lack access to advanced care.

Additional contributing factors to suboptimal care include limited health literacy, inconsistent follow-up, distance from specialized centers, and broader systemic constraints [8]. Insurance status and referral practices further affect access to advanced diagnostics and standard therapies [9,10]. Socioeconomic conditions influence the entire care continuum, from early symptom recognition to timely treatment and access to supportive care. These patterns persist even in well-resourced health systems [8,11].

The 2020 estimates from Global Cancer Observatory (GLOBOCAN) reveal that brain and central nervous system (CNS) cancer ranks 19th in global cancer prevalence (1.9% of all cases) and 12th in cancer mortality (2.5% of all cancer deaths) [12]. Globally, the age-standardized incidence of primary malignant brain and other CNS tumors in 2020 was approximately 3.5 per 100,000, with higher rates in males and in higher-income regions [13].

In the US, data from the Central Brain Tumor Registry of the United States (CBTRUS) for 2015–2019 reported an average annual age-adjusted incidence rate of 24.71 per 100,000 population for primary brain and other CNS tumors, with a higher prevalence in females compared to males (27.62 versus 21.60 per 100,000, respectively) [5]. This included 6.89 per 100,000 for malignant tumors and 18.46 per 100,000 for non-malignant tumors [5]. In contrast, the global incidence rate for primary malignant brain and CNS tumors in 2020 was 3.5 per 100,000, higher in males and high-income countries [5,13]. These results indicate that enhanced diagnostic capabilities and better healthcare access in high-income countries likely contribute to the higher reported incidence rates. Higher reported incidence in high-income countries likely suggests better diagnostics and access [14].

In the US, the incidence rates of brain tumors vary by age, the highest being in adults > 40 years and lower in children (0–14 years) and adolescents (15–19 years) [15]. There is also a notable correlation with race and SES, with higher rates observed in white individuals and those with higher SES [15,16]. The Global Burden of Disease Study in 2016 reported approximately 330,000 new cases of CNS tumors worldwide, a 17.3% increase since 1990 [17]. This increase was particularly notable in regions with a high Socio-demographic Index (SDI), such as Southeast Asia and Western Europe, with the most cases reported in China, the US, and India [17]. Results from this study highlight a strong association between higher SDI and increased CNS tumor incidence, indicating the influence of socio-demographic factors on their prevalence.

The CONCORD-3 study, part of the global CONCORD program, analyzed survival rates for various cancers, including brain tumors (2000–2014). Results from this study found that children generally have better survival rates than adults, with significant global variations [18]. This study also revealed differences in adult brain tumor survival by histology, with 5-year survival rates, in 2010–2014 subgroup patients, ranging from 20% to 38% for diffuse and anaplastic astrocytoma, 4% to 17% for glioblastoma (GBM), and 32% to 69% for oligodendroglioma [19]. These findings emphasize substantial global variation in the burden and survival rates of brain and CNS tumors, with outcomes differing markedly by age group and tumor histology.

Drawing on these global and national patterns, this review synthesizes evidence on diagnostic delays, treatment gaps, and survival disparities, with a primary focus on the US and selective international comparisons to contextualize US patterns.

## 2. Gaps in Diagnosis

Underserved groups have historically been associated with later presentation of brain tumors. Notable factors contributing to delayed presentation include low insurance coverage, health literacy, accessibility, affordability, and the availability of diagnostic healthcare. Delays in brain tumor diagnosis stem from inequity in access to healthcare. Hispanic individuals are 40% less likely to see a neurologist in an outpatient setting than non-Hispanic patients, while adjusting for insurance status, healthcare status, and income. Similarly, Black patients are 30% less likely to see a neurologist than White patients [20]. Several studies reveal the impact of SES on early diagnosis of brain tumors. Brewster et al. queried the Surveillance, Epidemiology, and End Results (SEER) and National Cancer Institute (NCI) databases to find that higher SES was linked to early diagnosis and younger age at diagnosis for intracranial meningiomas [21]. Similarly, they reported a higher propensity for complete resection and greater survival in patients with higher SES [21]. Diagnostic uncertainty is also seen more often in non-Hispanic Black patients, who are more likely to be diagnosed with brain lesions of unknown size and grade [22]. These data are important since timely access to neurological evaluation and diagnostic imaging is inequitably distributed. Early diagnosis is important in high-grade tumors where even short delays can significantly influence surgical candidacy and survival. The 2021 WHO classification further amplifies these disparities, as accurate diagnosis now relies heavily on molecular characterization [23]; limited access to next-generation sequencing and molecular pathology results in misclassification and delayed or inappropriate treatment, creating inequities at the very start of the diagnostic pathway. Moreover, diagnostic delays are also influenced by the inherent difficulty of distinguishing brain tumors from non-neoplastic conditions. Omuro et al. highlight that several inflammatory, infectious, and vascular diseases can closely mimic tumors on MRI or even histology, leading to misdiagnosis and delayed treatment [24].

Survival patterns also underline the influence of SES on brain tumor identification. Patients with higher SES benefit from better access to advanced diagnostics, including next-generation sequencing (NGS) and specialized care, leading to earlier diagnosis and broader treatment options. In contrast, patients with lower SES often experience delayed diagnoses and limited access to advanced technologies [25,26,27].

Beyond individual- and system-level barriers within the U.S., large-scale international patterns also reflect gaps in diagnostic capability and disease reporting. Global variations in brain and CNS tumor incidence are shaped by limitations in healthcare infrastructure and cancer registry systems [28]. In regions with restricted access to advanced diagnostic technologies like magnetic resonance imaging (MRI) and computed tomography (CT) scanners, underdiagnosis is common, particularly in low- and middle-income countries [29]. For example, Africa’s cancer registry coverage is limited to 31% of its geography and only 1% of its population, significantly affecting reported incidence rates [30]. Higher incidence rates of malignant brain tumors among Asians in Western countries compared to their native countries highlight the combined impact of genetic factors and healthcare access, and the need for genome-wide association studies (GWASs) to understand genetic and environmental influences on CNS tumor incidence and characteristics [30,31,32,33]. As a result, brain tumor burden in these regions may be significantly underestimated, limiting targeted public health interventions and allocation of resources for neurologic oncology services.

Barriers to timely diagnosis are further magnified in low- and middle-income countries. A 2024 systematic review by Shakir et al. reported that early detection is hindered by limited diagnostic infrastructure (38%), misdiagnosis by healthcare providers (33%), financial constraints (46%), and insufficient awareness of early warning signs among clinicians (21%) [34]. The lack of MRI and CT availability, weak referral systems, and absence of local clinical guidelines also impede timely evaluation. Proposed strategies include provider education initiatives (50% of studies), establishing streamlined referral pathways (25%), and implementing context-adapted diagnostic protocols (19%). Notably, structural constraints, rather than tumor biology alone, drive late presentation.

**Figure 1:**
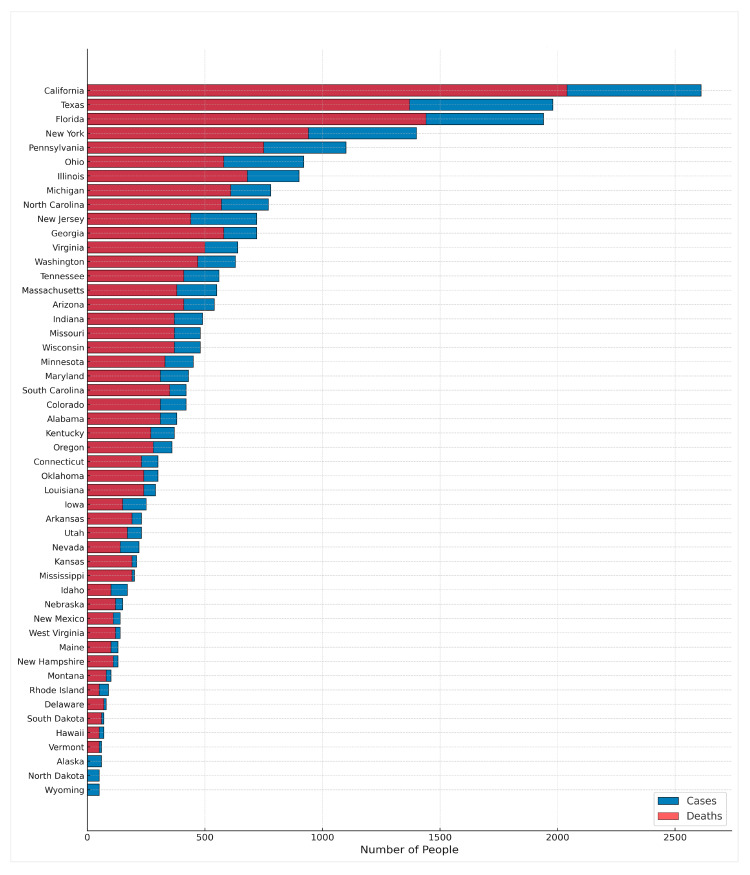
Estimated new cases and deaths for brain and other nervous system cancers in the United States, 2025, by state. Open-source data (Table S1) was obtained from the American Cancer Society (2025) [2].

Geographic differences in the US are highlighted in Figure 1, which displays the estimated new cases and deaths for brain and other nervous system cancers across the US in 2025.

In addition to structural and socioeconomic barriers, differences in tumor incidence may also reflect underlying genetic or biological variations among populations. Genomic studies suggest that European ancestry may increase glioma risk in adults [35]. Statistics from 2021 show that malignant brain tumors are more common in males and non-Hispanic Whites, while non-malignant tumors are more prevalent in females and non-Hispanic Black individuals. Marked differences in diagnostic timing and tumor classification remain, particularly in childhood tumors like diffuse astrocytoma (75% vs. 86%) and embryonal tumors (59% vs. 67%) for Black versus White patients diagnosed between 2009 and 2015 [36]. Equitable access to diagnostic tools, specialized neuropathological review, and genetic testing are all critical to ensure accurate tumor classification and timely intervention.

## 3. Gaps in Treatment

Treatment for brain tumors can be influenced by socioeconomic, geographic, and racial factors (Table 1), leading to unequal access to effective therapies. Patients with lower income, limited education, or inadequate insurance are significantly less likely to receive advanced treatment modalities. Additionally, the high costs of molecular diagnostics and targeted therapies further widen treatment inequities, as many patients cannot access these modalities even when clinically indicated [37]. Among patients with brain metastases, those with lower income, limited education, or inadequate insurance are significantly less likely to receive advanced treatment modalities. For instance, patients in the lowest socioeconomic quintile were 17% less likely to receive radiation and 35% less likely to receive chemotherapy [38]. Similarly, meningioma patients in lower SES groups are 24% less likely to undergo gross total resection [39]. Even within universal healthcare systems, significant differences remain. A Swedish study in 2020 found that low-income and less-educated glioma patients faced longer wait times and poorer pre-operative conditions despite universal coverage [40]. These findings suggest that financial status and health literacy continue to play a role even when cost barriers are theoretically minimized.

Geographic location also plays a substantial role in determining access to optimal treatment. In the US, rural regions often have fewer specialized healthcare providers and facilities, resulting in reduced access to neurosurgical expertise and cutting-edge therapies [41,42,43]. SEER data revealed a 15% higher mortality risk for rural brain cancer patients compared to their urban counterparts (HR = 1.15, 95% CI: 1.01–1.31) [6,44]. Contributing factors include facility closures, transportation barriers, and delayed referrals [44]. These structural limitations significantly impact treatment planning and access to timely interventions. In addition, a recent national assessment of neuro-oncology trial-supporting infrastructure reported that more than half of the US population lacks direct access to oncology clinical trial infrastructure, with over 70% of sites clustered in urban and socioeconomically advantaged areas, further constraining access to multidisciplinary care and novel therapies [45].

Racial and ethnic differences in treatment allocation remain a critical issue. African American and Hispanic patients are less likely to receive advanced treatments such as stereotactic radiosurgery for brain metastases compared to non-Hispanic White patients, often due to being treated at lower-volume centers or lacking referral to specialty care [3,9,46]. Kehm et al. observed that African American, Hispanic, and Asian patients received fewer supportive medications, including antiemetics, steroids, sleep aids, and appetite stimulants, than non-Hispanic White patients [10]. Such prominent gaps in supportive care further compound treatment burden and quality of life in underserved populations. Beyond medication use, the current literature shows several disparities. Minoritized patients more often present with greater premorbid illness and longer symptom duration. They also receive less information during clinical encounters, experience higher perioperative morbidity [47,48].

Differences in treatment patterns, including delays in diagnosis and therapy initiation, have been well-documented. Black and Hispanic White patients with brain metastases experience significant delays in starting treatment compared to non-Hispanic White patients [49]. National database studies reveal that Black patients with primary and metastatic brain tumors are less likely to be offered aggressive surgical intervention, and that non-White, non-Hispanic female patients are less likely to receive standard-of-care therapies for GBM [50,51]. Uninsured patients are also less likely to receive comprehensive treatment plans. Delays extend into the postoperative period, where Black patients tend to experience longer hospital stays and are more often discharged to non-home settings (e.g., skilled nursing facilities or inpatient rehabilitation centers) compared to White patients [52,53]. Furthermore, Black patients face prolonged delays in initiating radiotherapy after surgery for unresected GBM and both non-Hispanic Black and Hispanic White patients are less likely to receive radiation and chemotherapy overall [54,55]. Complementing these findings, a recent SEER-based study of 16,682 adults with GBM eligible for systemic chemotherapy after surgery and radiotherapy showed that omission of chemotherapy, although infrequent overall, was significantly more common among patients aged ≥80 years, those from lower-income areas, and those identifying as Hispanic, Black, or Asian/Pacific Islander [56]. These disparities in access to and delivery of timely, guideline-concordant care likely contribute to poorer outcomes for racial and ethnic minority patients with brain tumors.

Insurance status, proximity to research institutions, and lack of physician referral are all key contributing barriers that limit access to clinical trials and novel treatment opportunities for marginalized groups. Patients with health insurance are substantially more likely to participate in clinical trials compared to their uninsured counterparts. Financial toxicity, transportation issues, and trial design limitations further prevent individuals from accessing novel therapies [57,58,59]. Studies indicate that 42% of brain tumor patients are informed about available trials, and only 24% are informed at diagnosis with only 21% enrollment [57,60,61]. Physician-patient communication, linguistic differences, and low health literacy further reduce enrollment. Despite federal mandates under the National Institutes of Health Revitalization Act (1993), women comprise just over one-third of participants in CNS tumor trials, and racial/ethnic minorities represent only about 8%, a proportion that has not improved meaningfully over time [62].

These data collectively suggest that effective treatment for brain tumors is not currently equitably accessible. Structural, financial, and institutional barriers continue to affect whether patients receive timely, evidence-based care. Addressing these gaps is essential to ensure that all individuals have access to appropriate therapies, supportive care, and potentially life-prolonging clinical trials.

**Table 1:** Summary of key determinants influencing brain tumor treatment access and delivery.

Determinant	Mechanism	Impact on Treatment	References
Socioeconomic Status	Income, education, neighborhood disadvantage; financial toxicity and employment loss	Reduced access to surgery, RT, chemotherapy; omission of systemic therapy; delays in care	[38,39,40,56]
Insurance Coverage	Type and adequacy of insurance	Influences multimodal therapy and timeliness of RT	[63,64]
Geographic Location	Rurality; distance to specialty centers; uneven distribution of trial-supporting infrastructure	Limited neurosurgical access; higher mortality	[6,44,45]
Race/Ethnicity	Structural inequities; referral patterns	Lower likelihood of aggressive or timely treatment; higher perioperative morbidity; lower rates of molecular testing	[49,50,51,55,62]
Health Literacy	Ability to navigate diagnosis and treatment	Delayed presentation; reduced adherence	[20,21,22,25,26,27]
Referral Pathways	Variation in provider referrals and facility type	Affects access to surgery, SRS, advanced care	[9,49,50,51]
Access to Advanced Modalities	Institutional capability	Unequal access to genomic testing and treatments such as SRS, TTFields	[10,28,29]
Clinical Trial Access	Structural and logistic barriers; limited infrastructure; provider bias; inadequate outreach	Low enrollment among minorities, uninsured, and rural patients; limited access to novel therapies	[45,57,58,59,60,61,62,65]
Supportive Care Infrastructure	Availability of medications, rehabilitation	Impacts QoL, symptom burden, and treatment tolerance; disparities in access to brain tumor–specific palliative care and rehabilitation	[10,52,65]

Abbreviations: RT: radiotherapy; SRS: stereotactic radiosurgery; TTFields: Tumor Treating Fields; QoL: quality of life.

## 4. Gaps in Outcomes

SES has also been shown to impact brain tumor outcomes. A study analyzing data from 28,952 GBM patients diagnosed between 2005 and 2016 found that a higher median household income was associated with lower non-GBM mortality, while race and socioeconomic factors influenced both GBM-specific and other-cause mortality [66]. In another study, privately insured patients experienced lower short-term mortality compared to Medicaid recipients. These outcome differences are largely attributed to enhanced access to advanced diagnostics, specialized care, and comprehensive treatments such as surgery, radiation, and chemotherapy among individuals with higher SES [11]. Furthermore, individuals with higher SES often reside in healthier environments and benefit from lifestyle factors that contribute to improved overall health and cancer prognosis [67]. Table 2 highlights key studies demonstrating the association between SES indicators in GBM.

**Table 2:** Association between socioeconomic indicators and outcomes in glioblastoma.

Study	Study Type	Population	SES Measure	Key Findings
Di Nunno et al., 2024 [68]	Systematic Review/Meta-Analysis	143,303 GBM patients	Economic income	Lower economic income is associated with poorer survival (pooled HR 1.09, 95% CI: 1.02–1.17)
Estevez-Ordoñez et al., 2023 [69]	Retrospective Cohort Study	995 GBM patients (2008–2019)	Patient household income level (categorized low/middle/high); Insurance type; (also analyzed race)	African Americans had longer overall survival (aHR = 0.37) but significantly worse outcomes in low-SES groups (uninsured Black vs. White patients, HR = 15.6)
Ramapriyan et al., 2023 [70]	Retrospective Cohort Study	29,609 GBM patients	Race and social determinants of health	Non-White patients predominantly composed the lowest SES quartile; higher SES is associated with better access to care and outcomes
Dy et al., 2022 [71]	Systematic Review/Meta-Analysis	2552 GBM patients in low- and middle-income countries	Country income level	Patients in low- and middle-income countries have shorter survival times compared to those in high-income countries
Rivera Perla et al., 2022 [72]	Retrospective (multi-center) Study	434 adult GBM patients (2012–2017)	ADI: neighborhood SES composite (top 66% vs. bottom 33% percentile)	GBM patients from high-ADI areas had lower odds of gross total resection (aOR = 0.43) and reduced use of adjuvant therapies, though survival differences were not significant
Bower et al., 2020 [73]	Retrospective (Single center) Study	312 high-grade glioma patients (1999–2017)	Residential community income level (ZIP code median income above vs. below state median)	Patients from low-income areas had shorter OS (15.6 vs. 19.7 months) and living in high-income communities reduced mortality risk by 25% (HR = 0.75, *p* < 0.05) after adjustment
Liu et al., 2020 [66]	Retrospective Cohort Study	28,952 GBM patients	Median household income	Higher median household income is associated with better overall survival; racial differences also influence outcomes
Ostrom et al., 2018 [74]	Retrospective Cohort Study	244,808 glioma patients of which 150,631 (61.5%) had GBM	Race/Ethnicity	Non-Hispanic whites have higher glioma incidence, but lower survival rates, compared to other racial/ethnic groups
Pollom et al., 2018 [63]	Retrospective Cohort Study	12,738 adult GBM patients (2010–2013) with chemoradiation	Area-level median household income (by ZIP code; above vs. below $48,000/year); Insurance status	Lower-income and uninsured/Medicaid patients had delays >35 days in starting radiotherapy, which correlated with worse survival. Patients from higher-income areas were more likely to receive timely RT (within 35 days) after surgery, conferring a survival benefit
Rong et al., 2016 [64]	Retrospective (SEER analysis) Study	13,665 adult GBM patients (2007–2012)	Insurance status (Uninsured, Medicaid vs. non-Medicaid insured)	Uninsured (HR = 1.14) and Medicaid (HR = 1.10) patients had shorter survival than privately insured ones, with better survival outcomes over time seen only among the insured

Abbreviations: GBM = glioblastoma; SES = socioeconomic status; ADI = Area Deprivation Index; OS = overall survival; HR = hazard ratio; aHR = adjusted hazard ratio; aOR = adjusted odds ratio; RT = radiotherapy; CI = confidence interval.

Internationally, outcome variation is equally striking. Brain tumor outcomes are generally worse in countries with lower SES and less advanced healthcare systems [65,75]. Minority groups with primary CNS tumors face significantly elevated mortality risk, particularly in high-grade gliomas [9]. For example, lower SES accounts for 33.7% of the increased hazard rate in non-Hispanic Black children, who still experience higher mortality than White children even after adjusting for SES [9]. Glioma incidence is also higher in high-income countries, likely reflecting superior diagnostic capabilities and healthcare access, while patients in low- and middle-income countries often remain underdiagnosed or untreated [17,75,76].

Regional data within the US demonstrate that outcomes also vary by geography. A SEER-based study analyzing data from 2004 to 2013 showed that the Southern US region had the highest GBM incidence (24.31 per 100,000/year) and mortality, followed by the Northeast (22.6), West (20.35), and North Central regions (15.03) regions [77]. Even though incidence was high, mortality remained elevated in the South, suggesting that geographic access to care and follow-up services remains a key determinant of survival. Rural regions face persistent structural disadvantages. SEER data revealed a 15% higher mortality risk for rural brain cancer patients compared to their urban counterparts [6]. Similar trends have been observed in Appalachia [7,44,78], China [79], and South Korea [80]. Higher urban mortality has been noted in India, likely due to cultural and healthcare delivery factors, though inconsistencies in cancer reporting may also play a role [81].

Global trends mirror these regional observations. The Global Burden of Disease Study (1990–2016) reported a 17.3% increase in age-standardized incidence rates of brain and CNS tumors, with approximately 330,000 new cases and 227,000 deaths by 2016 [17]. Higher incidence rates were concentrated in high SDI regions such as East Asia, Western Europe, and countries like China, the U.S., and India. While age-standardized disability-adjusted life-years (DALYs) decreased in high SDI regions, they increased in lower SDI regions, indicating widening outcome gaps. Wrensch et al. further highlighted international variation in malignant brain tumor incidence, with rates in countries like Australia, Canada, and the U.S. up to four times higher than those in Rizal, Philippines, and Mumbai, India [41]. Collectively, these patterns highlight that brain tumor outcomes are shaped as much by geography and healthcare infrastructure as by tumor biology, highlighting the urgent need to address structural disparities in diagnosis and treatment worldwide.

The influence of social support and psychosocial stability on outcomes has also been well-documented. Brain tumor diagnosis and treatment significantly affect physical, cognitive, and emotional functioning. Patients from lower SES backgrounds face greater psychological and cognitive challenges. In pediatric brain tumor patients, higher SES correlated with significantly better outcomes, including a 17-point IQ and 13-point math score advantage over low SES counterparts before radiotherapy; nearly one standard deviation in magnitude [9]. Additionally, 25% of the variation in psychological stress index (PSI), linked to quality of life, was attributed to SES differences [6]. Patel et al. noted that parent education and child motivation were among the key socio-ecological factors reducing health-related quality of life by up to 25% [14].

Individual resilience and social support networks also influence outcome trajectories. Patients with strong family support demonstrate better treatment adherence, lower rates of depression and anxiety, and overall improved quality of life [16,17,18]. In contrast, social isolation is associated with worse outcomes. Glantz et al. found that divorced or separated patients were more likely to experience higher hospitalization rates, lower clinical trial participation, and reduced access to treatments such as cranial irradiation. These patients were also less likely to die at home, pointing to significant deficits in emotional, logistical, and financial support. The absence of a partner or caregiver ultimately translated into poorer quality-of-life and end-of-life outcomes (*p* < 0.0001) [75].

## 5. Addressing Existing Gaps and Future Directions

Accumulating evidence suggests that brain tumor outcomes are inextricably linked to broader social determinants, including healthcare accessibility, geographic gaps, and SES.

The findings presented across diagnosis, treatment, and outcomes show that patient access to timely care, advanced diagnostics, and effective therapies is uneven. Differences in insurance coverage, geography, and healthcare infrastructure affect how patients navigate the care pathway and what services they ultimately receive. These factors also influence quality of life and survival, particularly in those with high-grade brain tumors.

Improving access requires changes across policy, clinical practice, and health system delivery. Reducing out-of-pocket costs and improving insurance coverage are key steps, especially for patients who face delays due to financial limitations. Increasing diversity in the healthcare workforce may improve communication and trust between patients and providers, which can help with clinical trial participation and treatment adherence [82].

Several barriers remain, including limited availability of specialists in rural areas, long travel distances to academic centers, and differences in provider referral patterns. Practical solutions such as telemedicine, regional partnerships between hospitals, travel support, and investments in local infrastructure can help close these gaps.

There is a need for more research that evaluates how social and economic factors affect brain tumor care and outcomes. Studies should also examine which interventions are most effective at improving access and how recent policy changes are affecting care delivery. While new therapies and diagnostic tools continue to emerge, it is important that patients have access to these advances.

Several policy documents provide guidance. The 2022 Agency for Healthcare Research and Quality (AHRQ) Report, the American Association for Cancer Research (AACR) Cancer Progress Report, and the 2020 American Society for Clinical Oncology (ASCO) Equity Statement emphasize access to high-quality care, workforce training, and stronger community partnerships [83,84,85]. The “Elevating Cancer Equity” report by the National Comprehensive Cancer Network (NCCN) calls for improvements in genetic testing access, trial enrollment, patient navigation, and consideration of non-medical factors that influence care [86].

Improving care for all patients with brain tumors will depend on systems that support early diagnosis, consistent treatment, and long-term follow-up.

## 6. Conclusions

This review outlines key challenges in brain tumor care in the United States linked to race, SES, and geography. Differences in access, diagnosis, and outcomes reflect broader gaps in healthcare delivery. Addressing these requires policy reform, tailored treatment strategies, and expanded tools such as telemedicine and workforce development. Advances in technology and precision medicine offer new possibilities but must be paired with patient-centered approaches that reflect cultural and individual needs. Collaborative efforts across systems are essential to improve care and outcomes for all brain tumor patients.

## Supplementary Materials

The following supporting information can be downloaded at: https://www.mdpi.com/article/10.3390/cancers17243982/s1, Table S1: Estimated New Cases and Deaths for Brain and Other Nervous System Cancers in the United States, 2025, by State.

## Abbreviations

The following abbreviations are used in this manuscript:

AHRQ	Agency for Healthcare Research and Quality
ASCO	American Society of Clinical Oncology
CBTRUS	Central Brain Tumor Registry of the United States
CNS	Central Nervous System
DALY	Disability-Adjusted Life Year
GBM	Glioblastoma
GLOBOCAN	Global Cancer Observatory
MRI	Magnetic Resonance Imaging
NCCN	National Comprehensive Cancer Network
NGS	Next-Generation Sequencing
PSI	Psychological Stress Index
SDI	Socio-demographic Index
SEER	Surveillance, Epidemiology, and End Results
SES	Socioeconomic Status
