# Supplementary material for: Gaps in Diagnosis, Treatment, and Outcomes Among Patients with Brain Tumors in the United States: A State-of-the-Art Review
Source: Cancers (Basel). 2025 Dec 13;17(24):3982. doi: 10.3390/cancers17243982 (PMC12730981; doi:10.3390/cancers17243982)
Supplement: Supplementary file 1 [file cancers-17-03982-s001.zip › cancers-4028967-supplementary.pdf]

Supplementary Table S1: Estimated New Cases and Deaths for Brain and Other Nervous System Cancers in the United States, 2025, by State.

State	Cases	Deaths
Alabama	380	310
Alaska	60	
Arizona	540	410
Arkansas	230	190
California	2,610	2,040
Colorado	420	310
Connecticut	300	230
Delaware	80	70
Florida	1,940	1,440
Georgia	720	580
Hawaii	70	50
Idaho	170	100
Illinois	900	680
Indiana	490	370
Iowa	250	150
Kansas	210	190
Kentucky	370	270
Louisiana	290	240
Maine	130	100
Maryland	430	310
Massachusetts	550	380
Michigan	780	610
Minnesota	450	330
Mississippi	200	190
Missouri	480	370
Montana	100	80
Nebraska	150	120
Nevada	220	140
New Hampshire	130	110
New Jersey	720	440
New Mexico	140	110
New York	1,400	940
North Carolina	770	570
North Dakota	50	
Ohio	920	580
Oklahoma	300	240
Oregon	360	280
Pennsylvania	1,100	750
Rhode Island	90	50
South Carolina	420	350

South Dakota	70	60
Tennessee	560	410
Texas	1,980	1,370
Utah	230	170
Vermont	60	50
Virginia	640	500
Washington	630	470
West Virginia	140	120
Wisconsin	480	370
Wyoming	50	
